# Peritonitis Due to Appendicitis Related to Mercury Sequestration: An Unusual Peruvian Case Report

**DOI:** 10.7759/cureus.27667

**Published:** 2022-08-04

**Authors:** José Casas Roca, Anthony Ramos-Yataco, Carlos Alcalde-Loyola, Gandhy Montalvo, Jeniffer Rios-Rojas, Alejandra Bacilio Cardozo

**Affiliations:** 1 Pathology, Pathology Service, Hospital II-1, Moyobamba, PER; 2 Internal Medicine, Hospital Ricardo Cruzado Rivarola, Nasca, PER; 3 Internal Medicine, Universidad Nacional de Trujillo, Trujillo, PER; 4 Internal Medicine, Federico Villarreal National University, Lima, PER; 5 Internal Medicine, National University of San Marcos, San Fernando Medical School, Lima, PER; 6 Surgery, General Surgery Service, Hospital II-1, Moyobamba, PER

**Keywords:** sequestration, peruvian, peritonitis, appendicitis, mercury

## Abstract

Elemental mercury ingestion caused by folk practices is rare and usually harmless. Nevertheless, some complications related to mercury ingestion have been reported such as appendicitis related to mercury sequestration and poisoning leading to systemic toxicity. Patients usually present with nausea, vomiting, and abdominal tenderness. Mercury sequestration in the appendix depends on its anatomy and mercury physical properties, both of which may lead to appendicitis, resulting in subsequent peritonitis leading to multiple and severe surgical complications. A 26-year-old Peruvian man complaining of vomiting and abdominal pain after ingestion of elemental mercury as part of a folk practice presented to the emergency department. Physical exam was remarkable for rigid abdomen and diffuse rebound sign. A clinical diagnosis of peritonitis was made. The patient was taken to the operating room where an open appendectomy and peritoneal lavage were performed. On gross inspection, a silver foreign body within the perforated appendix was seen by the surgical team. The patient developed multiple surgical complications leading to multiple organ failure and death. Clinicians should be aware that mercury ingestion is usually benign. However, severe complications may develop. Early surgical and medical intervention should be initiated promptly to achieve better outcomes. We present the first case of peritonitis due to appendicitis related to mercury sequestration in the appendix.

## Introduction

In the last decade, the rate of mercury intoxication has increased globally. People can get intoxicated accidentally or intentionally [1].

In Peru, the main cause of mercury exposure is related to illegal gold mining, which uses large amounts of elemental mercury. Elemental mercury forms organic mercury, which becomes part of the food chain. Therefore, organic mercury is the main form of intoxication [2-3]. In contrast, people’s exposure to elementary mercury typically only occurs through its use in folk healing practices [3-4].

Mercury can be found in three forms: elemental mercury, inorganic mercurial salts, and organic mercury. They differ in properties but all of them are toxic [1,5]. The elemental mercury is a silvery liquid at room temperature. In contrast to the other forms, elemental mercury is poorly absorbed by the gastrointestinal (GI) tract after ingestion [3]. Hence, its consumption is considered less toxic. Nevertheless, small amounts of elemental mercury can remain in the GI tract, causing nausea, vomiting, abdominal pain, bloody diarrhea, and potential necrosis of the GI tract mucosa [5]. In extremely rare cases, mercury may be sequestered in the appendix, which contributes to the pathophysiology cascade for appendicitis and is an extremely rare cause of peritonitis [6-7].

We describe the case of a young man who ingested elemental mercury as a part of a folk remedy to heal his GI condition. The elemental mercury may have become sequestered in the appendix causing appendicitis leading to peritonitis and multiple complications. This is a unique case that contributes to a new possible life-threatening complication from mercury elemental ingestion.

## Case presentation

**Figure 1:**
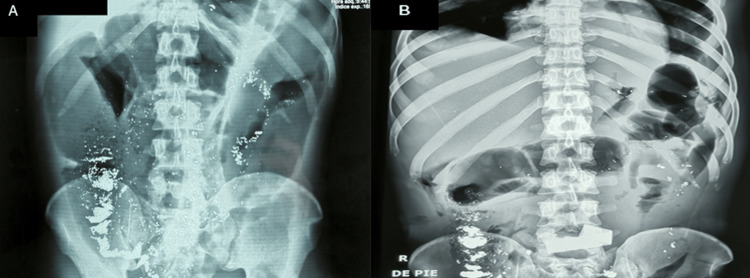
Abdominal X-ray (A) X-ray taken at hospital admission (second day after mercury ingestion). It shows several radiopaque particles throughout the gastrointestinal tract, sequestration in the appendix, and elemental mercury throughout the peritoneal cavity. (B) X-ray taken on the sixth day after mercury ingestion.

A 26-year-old Peruvian man was presented to the emergency department (ED) because of abdominal pain and nausea without fever. He reported no prior relevant medical history. In addition, two days before admission, he indicated that he had consumed a treatment given to him by his folk practitioner that consisted of vegetable products and 1 ounce of elemental mercury. At the ED, vital signs were as follows: heart rate of 110 beats per minute (bpm), respiratory rate of 24 cycles per minute (cpm), blood pressure of 90/60 millimeters of mercury (mmHg), temperature of 98.4 Fahrenheit degrees (F^0^) an oxygen saturation of 96% on room air. Physical examination was remarkable for rigid abdomen and diffuse rebound sign. Laboratory blood tests showed a high white blood cell count of 20.89 x103/mm^3^ with 90% of neutrophils and 2% bands. Liver function test and amylase were within normal limits. C-reactive protein (CRP) was 41.5 mg/dL. An abdominal X-ray showed several metal-like radiopaque particles throughout the GI tract (Figure 1A). A suspected preoperative diagnosis of peritonitis was made. In the operating room (OR), the surgical team found 4 liters of fecaloid material in the peritoneal cavity, several adhesions between the intestinal peritoneum, and a 10 cm appendix with a 0.5 cm perforation at the base. It contained an atypical foreign body within its lumen. Appendectomy and adhesions release were performed. Furthermore, peritoneal lavage with abundant saline approximately retrieved 0.5 ounces of mercury. Laminar drainages were inserted in the peritoneal cavity and broad-spectrum antibiotics were started.

**Figure 2:**
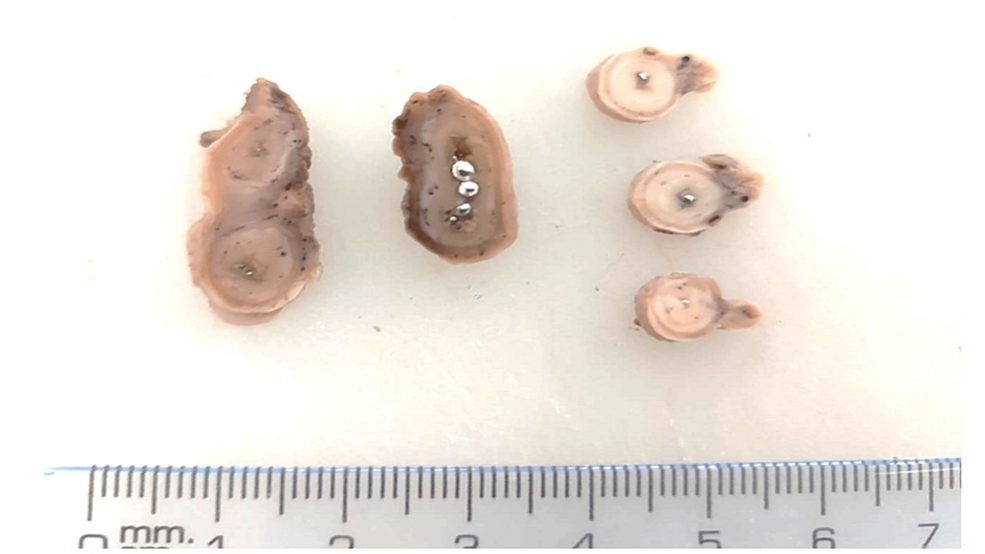
Specimen of the appendix after surgery Elementary mercury sequestered in the appendix lumen

On the second day after surgery, fecaloid discharge was noted in drains. The patient was taken to the OR, a right colic flexure rupture was found, and a colostomy was performed. Multiple drainages were put in place. Two days after the second surgery, the patient became hypotensive and physical examination showed a rigid abdomen and persistent fecaloid drainage. A new abdominal X-ray showed persistent metal-like radiopaque particles in the GI tract (Figure 1B). The patient underwent a third surgery that showed an ileum perforation; subsequently, an ileostomy was performed. Nevertheless, the patient had poor clinical evolution and developed acute kidney injury and liver dysfunction leading to multiple organ failure. He expired on the third day after the last surgery. Pathology of the appendix showed areas of necrosis and hemorrhage and the presence of retained mercury (Figure 2).

## Discussion

We report the first case of elemental mercury ingestion that occurred as a result of using a folk remedy, with subsequent retention of mercury in the appendix causing appendicitis that leads to peritonitis. Approximately 12 similar cases of mercury sequestration in the appendix and only three cases of appendicitis related to elementary mercury are found in the literature [7-10].

Mercury is used in folk practices in the form of inorganic salts, and it is relatively common within certain Hispanic communities [8,11]. Pure elemental mercury in liquid form is often used in folk remedies to treat abdominal pain and other illnesses [12-13].

In the present case, our patient ingested approximately one ounce of liquid mercury that was provided by a folk practitioner for the purpose of curing abdominal illness. It is known that mercury toxicity occurs via the inhalatory route from mercury vapor. Acute elemental mercury ingestion is considered benign because systemic absorption is unlikely. This is mainly due to less than 1% absorption of elemental mercury from the gastrointestinal tract [14]. Furthermore, elemental mercury is mostly eliminated in feces days after ingestion. Systemic toxicity is rare and generally not expected [15-16]. Only two cases of systemic intoxication after massive ingestion have been reported [17]. It is hypothesized that mercury can form globules in the GI tract that can release vapor at body temperature [18]. Up to 80% of this mercury vapor can be absorbed by the lungs and diffuse into the bloodstream, which leads to absorption by other organs, thereby causing neurological and renal toxicity [19].

The occurrence of sequestration of mercury in the appendix depends not only on the anatomic characteristics of the appendix lumen, which can be wide open and vertical, but also on the weight of mercury, which is greater than the bowel fluid contained in the lumen. Any of the foregoing factors can contribute to mercury being arrested in the cecum during transit and gravitating toward the lower portion of the appendix. In this location, peristalsis motion is usually insufficient to expel the mercury thereby causing sequestration [7,20]. As a result, elementary mercury may remain immobile in the appendix, stimulating an inflammatory reaction with the possibility of perforation leading to peritonitis [5]. Another important factor in our case is the role of mercury retained in the GI tract for a prolonged period of time, which permits the bacterial conversion of elemental mercury to organic mercury leading to toxicity [20].

Several studies have revealed that mercury exposure produces damage and serious intestinal disease, including damage to tight junction proteins and necrosis of epithelial cells through an inflammatory response [21]. Research studies in mice showed that five days after exposure to inorganic mercury, the GI mucosa increased the expression of inflammatory cytokines, such as tumor necrosis factor-alpha (TNF-α) and interleukin six (IL-6), in both the duodenum and colon. In addition, mercury may cause hemorrhagic colitis and disruption of the intestinal barrier related to gut immunity and oxidative stress induced by mercury exposure [22]. Our patient developed rapidly progressive proximal bowel perforations. We hypothesize that inflammation and contamination from appendix rupture, probable secondary infection, elemental mercury as an aggravating factor, and increased intestinal oxidative stress caused an inflammatory response that affected the proper healing of the gastrointestinal mucosa, all of which may have led to colonic and ileum perforations leading to feculent discharge in the peritoneal cavity. Furthermore, complicated appendicitis may cause entrapped fecal material in the bowel, leading to increased intraluminal pressure, stasis, inflammation, and associated infection leading to marked frank perforation.

The proper management protocol of mercury sequestration in the appendix is in debate. Many specialists choose prophylactic appendectomy, with the intention to avoid complications from an obstructed appendix. For example, the lumen can become inflamed and perforated, resulting in the possible effusion of mercury into the peritoneal cavity, thereby causing subsequent systemic toxicity and peritonitis [18]. On the other hand, a conservative approach that includes gastric lavage, cleaning up via motility, and use of decompression colonoscopy helps with the elimination [20,23-24]. Additionally, the left lateral decubitus positioning has been successfully employed and offers a potential alternative [25]. Our surgery team took the decision to not perform a decompression colonoscopy to try to evacuate mercury from the colon of the patient because of the patient`s critical state. Systemic toxicity may be treated with chelating agents such as unithiol (2,3-Dimercapto-1-propanesulfonic acid) and succimer (dimercaptosuccinic acid) [9,26]. However, the effectiveness of each management protocol (conservative or surgical) should be assessed on a case-by-case basis. Our case showed the complications of sequestration of mercury leading to appendicitis and subsequently peritonitis, thus highlighting the need to consider early surgical intervention and monitoring for evidence of mercury poisoning, which would require chelation therapy.

Treatment of free elemental mercury contamination in the peritoneal cavity has not been reported in the literature. Our surgical team took the decision to try to remove elemental mercury through peritoneal lavage without success. More research is needed to establish an adequate standard of care.

## Conclusions

In conclusion, elemental mercury ingestion may be sequestered in the appendix, which may cause appendix rupture and spillage of elemental mercury in the peritoneal cavity, thereby being an aggravating factor for the development of multiple complications such as peritonitis and bowel perforations. Early surgical management is essential to avoid several complications. Nevertheless, more research is needed to provide appropriate care to patients with these complications.
